# Veterinary Medicine

**Published:** 1837-01

**Authors:** 


### VETERINARY MEDICINE.

*On the Influence of the Epidemic Constitution upon Animals, observed during the prevalence of the Epidemic Cholera.* By THE FACULTY OF MEDICINE AT VIENNA.

THE reports sent from the different provinces of Lower Austria, Moravia, Galicia, and Bohemia, respecting the influence of the Cholera Epidemic on animals, and especially the domestic class were laid, by order of the *Imperial Government*, before the faculty of Vienna, for the purpose of establishing a judgment on the point. The medical college published the following remarks, dated February 14, 1834.

It has been proved by innumerable facts, that, during the reign of the epidemic, the general health of animals suffered in various ways, which indisposition was too generally spread and too closely connected with the epidemic character of the cholera to admit of its being traced to other accidental causes, or of its being considered unconnected with the prevailing epidemic constitution. Even the symptoms of the disease, and the pathological changes observed upon examining the animals after death, exhibited a striking similarity to those produced by the human cholera. Hence arises the important result: "that the Asiatic cholera is a purely epidemic disease;" that is, a disease depending upon a peculiar epidemic constitution highly injurious alike to men and animals.

Although this decision may appear objectionable to those who maintain that the disease depends purely on contagion, still opinions merely hypothetical must give way to the fixed laws of nature, and the incontrovertible experience founded upon them; and the absurdity of attributing this scourge of society to the opening of a letter or a sack of wool, must be apparent to any one who considers its irresistible progress. Although the peculiar agency is still problematical, yet it appears satisfactorily proved not to depend exclusively on the condition of the atmosphere, since animals that live in water only, as fish, crabs, leeches, &c. died in great numbers at the time of the cholera pandemic.

Amongst domestic animals, the influence of the epidemic constitution was less observed in those whose internal organization differed most from the human, and especially in the organs of digestion; for instance, the ruminating animals. As in this latter class vomiting cannot take place, so it is hardly possible that any affection bearing the form of pure cholera can be imagined. Dogs and cats frequently died under appearances of cholera; and hares particularly, among wild animals. But birds seemed to suffer most, and the disease was observed to bear a great similarity to the human cholera. Upon the whole, the meteorologic changes which took place during the epidemic diarrhœa, seemed to be particularly obnoxious to the constitution of birds; as the fact that sparrows, crows, and singing birds appeared very rarely, or not at all, was amongst the most common occurrences. Fish were observed to be affected particularly in those places where the cholera raged with the greatest virulence and where there were great inundations.

*Jahrb. d. Oest. Staates*, 1835.

